# A Contemporary Retrospective Study of Survival in Dogs With Primary Lung Tumors: 40 Cases (2005–2017)

**DOI:** 10.3389/fvets.2020.519703

**Published:** 2020-10-23

**Authors:** Ruth J. Rose, Deanna R. Worley

**Affiliations:** ^1^Department of Clinical Sciences, Colorado State University, Fort Collins, CO, United States; ^2^Flint Animal Cancer Center, Colorado State University, Fort Collins, CO, United States

**Keywords:** survival, metastasis, lymph node, canine, primary lung

## Abstract

**Objective:** To report the median survival time in a contemporary cohort of dogs with primary lung tumors and intrathoracic nodal metastasis.

**Design:** Retrospective Case Series.

**Animals (or sample):** Dogs with primary lung tumors treated with lung lobectomy and lymph node biopsy.

**Procedures:** The medical record database at Colorado State University was queried for dogs with primary lung tumors from January 1, 2005 to December 31, 2017. Patients were identified for inclusion if they had lung lobectomy and an intrathoracic lymph node biopsy performed. The median survival time (MST) for lymph node positive (LN+) and negative dogs (LN–) was calculated as well as the MST in dogs that did or did not receive adjuvant chemotherapy. Differences were compared between groups with significance set at *p* < 0.05.

**Results:** The MST in LN+ dogs (*n* = 11) was 167 days which was not statistically different from LN– dogs (*n* = 29) at 456 days (*p* = 0.2407). No significant difference in the MST in LN+ dogs was identified between dogs that received adjuvant chemotherapy (*n* = 4; 110 days) and those that did not receive adjuvant chemotherapy (*n* = 6; 125 days) (*p* = 0.4409). There was no difference in survival time in LN– dogs receiving chemotherapy (*n* = 12; 335 days) as compared to those LN– dogs (*n* = 10) that did not receive adjuvant chemotherapy (258.5 days; *p* = 0.6475).

**Conclusions and Clinical Relevance:** The survival of primary pulmonary neoplasia in dogs with intrathoracic nodal metastasis is longer than previously reported in this contemporary cohort. Chemotherapy did not appear to improve survival in LN+ or LN– dogs. The combination of tumor size between 100 and 999 cm^3^ and positive lymph node status significantly reduced survival.

## Introduction

Primary lung tumors in dogs have a relatively low incidence within the pet population occurring in ~2–4 dogs per ten thousand in the US and UK (1–5). It is a disease mostly of older dogs with an average age of 10–11 years and may be over-represented in the Boxer, Doberman Pinscher, Australian Shepherd, Irish Setter and Bernese Mountain Dog breeds, although this finding is not consistent across studies (5–9). Whereas, there is a clear relationship between cigarette smoking and the increased risk of developing lung cancer in people, no apparent environmental factors have been identified in dogs (10–12). The clinical signs in dogs are coughing in 50–93%, dyspnea, lethargy, anorexia, weight loss, hemoptysis, and lameness secondary to hypertrophic osteopathy or rarely metastatic lytic lesions (6, 9, 13–18). In up to 30% of cases, primary lung tumors are an incidental finding (6, 9, 13, 16).

Carcinomas comprise the majority of primary lung tumors with metastasis occurring via lymphatic pathways or hematogenously and intra-airway metastasis reported for pulmonary sarcoma (5, 7, 19, 20). The most common carcinomas reported are pulmonary papillary or bronchoalveolar carcinoma and adenocarcinoma (differentiated and non-differentiated) (9, 13, 21). Of the several prognostic indicators of survival identified, lymph node status, whether lymph nodes are positive (LN+) or negative (LN–) for metastasis, greatly impacts survival (13, 21–23). The diagnosis of a LN+ primary lung cancer garners a grave prognosis with a median survival of 60 days (Ogilvie *n* = 12), 26 days (McNeil *n* = 15), 58 days (Polton *n* = 16) and more recently, 131 days (Paoloni *n* = 6) as compared to 285 days for LN– dogs (13, 21–23). In addition, tumor grade, clinical signs at diagnosis, histologic diagnosis, completeness of tumor excision, and the size of the tumor have also been associated with survival (13, 21, 24). Given the notable difference in survival time when LN metastasis is present, a thoracic computed tomography (CT) study is encouraged as part of the diagnostic work-up for patients with primary lung tumors (25). CT imaging can detect smaller pulmonary parenchymal changes such as intra-parenchymal metastasis compared to radiography as well as identify the presence of tracheobronchial lymphadenopathy (23, 25–27). Tracheobronchial lymphadenopathy strongly associates with lymphatic metastasis and this may inform an owner's decision about whether or not to proceed with thoracic surgery for tumor removal (23). Based upon the literature, the presence of lymph node metastasis suggests a grave prognosis, however, our clinical impression is that these patients live longer following treatment.

Therefore, we performed a retrospective study to report the median survival time of a contemporary cohort of dogs with primary pulmonary neoplasia and intrathoracic nodal metastasis. Our hypotheses were that the median survival of LN+ dogs having primary pulmonary neoplasia would be greater than 60 days and that adjuvant chemotherapy would have no effect on survival in dogs with lymph node metastasis.

## Materials and Methods

### Case Selection Criteria

The medical records database at Colorado State University Veterinary Teaching Hospital was queried for all dogs diagnosed with a primary lung neoplasia between January 1, 2005 and December 31, 2017. Cases were included if a histologic diagnosis of primary pulmonary neoplasia was made and a lymph node biopsy with histopathology was performed at the time of surgery. Cases were excluded if histopathology confirmed a diagnosis of histiocytic sarcoma or mesenchymal neoplasia or if a secondary neoplasia was identified during treatment.

### Medical Records Review

For each dog, signalment, weight, sex, spay/neuter status, clinical signs at presentation, CT findings, tumor size by volume, surgical approach, lung lobe(s) removed, histologic diagnosis of primary tumor and lymph node, mitotic index and grade, histologic margins, adjuvant treatment and date of death were noted. Tumor volume was calculated when measurements were available in three dimensions and then categorized as <100 cm^3^, 100–999 cm^3^, or >1,000 cm^3^ as previously reported (24). The survival time for each individual was defined as the time from surgery until death from progressive disease. Patients lost to follow-up and those alive at the time of writing were censored at the last known interaction for median survival time (MST) analysis. The MST was calculated using a Kaplan Meier survival analysis for patients with positive lymph node metastasis, negative lymph node metastasis, and lymph node status with or without adjuvant chemotherapy. A Mantel Cox test was used to evaluate differences between survival with significance set at *p* < 0.05. A Cox Regression was performed to evaluate multiple variables related to survival time. A student's t test was utilized to assess differences in the age, gender, weight, and mitotic index between LN+ (positive lymph node metastasis) and LN– (negative lymph node metastasis) groups. All statistics were performed using commercially available software^1^^,^^2^.

## Results

### Case Selection Results

Four hundred and forty-eight cases of primary lung tumors were identified within the database with 98 of these having surgery for tumor removal. Of these 98 cases, 51 (52.04%) had a lymph node biopsy performed and 47 (47.96%) did not. Three patients were removed from the lymph node biopsy group because one patient had insufficient information regarding surgery and two others had multiple concurrent cancers resulting in a total of 48 lymph node biopsy patients. An additional five cases were excluded because of the diagnosis of histiocytic sarcoma, one was removed for the diagnosis of myxosarcoma, and two additional cases were removed due to a secondary neoplasia being identified (thymoma). For the patients that had a lymph node biopsy performed, 11 (27.50%) were found to be LN+ for metastasis and 29 (72.50%) were LN– for metastasis.

### Signalment, Clinical Presentation, and CT Results

LN+ breeds reported were mixed breed (*n* = 5; 45.45%), Labrador Retriever (*n* = 2; 18.18%) and one each (9.09% per breed) for German Short-haired Point, Australian Shepherd, Pomeranian, Staffordshire Terrier. LN– breeds included mixed breed dogs (*n* = 8; 27.59%), Labrador Retrievers (*n* = 5; 17.24%), Boston Terriers (*n* = 2; 6.90%), Lhasa Apso (*n* = 2; 6.90%), Staffordshire Terriers (*n* = 2, 6.90%), and one each (4.45% per breed) for Airedale, Australian Shepherd, Border Collie, Fox Terrier, Shih Tzu, Toy Poodle, English Setter, Doberman Pinscher, Greyhound, and Jack Russell Terrier. There were 8 (72.72%) spayed female LN+ dogs, 15 (51.72%) spayed female LN– dogs, 3 (27.27%) castrated male LN+ dogs, 13 (44.83%) LN– castrated male dogs, and 1 intact male dog with a negative LN biopsy (3.45%). The mean age of LN+ dogs was 11.97 ± 1.47 years (9.58–14 years) and was 11.30 ± 1.99 years for LN– dogs with a range of 7.75–14.92 years. There was no statistical difference in mean age between the LN+ and LN– dogs (*p* = 0.1879).

The most common clinical signs/presenting complaint for all dogs in the study was coughing (Table 1). Seven of 11 LN+ (63.64%) dogs had a CT scan performed wherein 3 (42.86%) were reported to have enlarged lymph nodes, 3 (42.86%) were reported to be normal and neither the report nor the images were available for review for one case (14.29%). Twenty-three of 29 (19.31%) LN– dogs had a CT scan and of these, 2 (8.70%) patients were reported to have enlarged lymph nodes, 21 (91.3%) were reported to be normal. Tumor volume was available for 7 LN+ (63.64%) cases. Volume was less than 100 cm^3^ for 0 LN+ cases, >100 and <999 cm^3^ for 6 (85.71%) and >1000 cm^3^ for 1 (14.28%) LN+ cases. For LN– cases, tumor volume was available for 14 (48.28%) cases wherein 7 (50.00%) were <100 cm^3^ and 7 (50.00%) were between 100 and 999 cm^3^.

**Table 1:** Presenting clinical signs.

	**LN+ (*n* = 11)**	**LN– (*n* = 29)**
**Clinical sign/complaint**		
Coughing	9 (81.82%)	19 (65.52%)
Wheezing	0	1 (3.45%)
Inappetence	1 (9.10%)	1 (3.45%)
Exercise intolerance	1 (9.10%)	1 (3.45%)
Lethargy	0	2 (6.91%)
Increased respiratory effort	1 (9.10%)	0
Gagging/retching	0	2 (6.91%)
Vomiting	0	1 (3.45%)
Panting	0	1 (3.45%)
Lameness	0	1 (3.45%)
Weight loss	0	0
Chronic upper respiratory disease	0	2 (6.91%)
Pneumonia	0	1 (3.45%)
None	0	4 (13.79%)

### Surgical Details

For primary pulmonary tumor removal, an intercostal approach was performed for 9 (81.82%) LN+ cases and 27 (93.10%) LN– cases whereas a median sternotomy was performed in 2 (18.18%) LN+ case and 2 (6.90%) LN– cases. The left cranial lung lobe was removed in 4 (36.36%) LN+ cases and 3 (10.34%) LN– cases, the left caudal was removed in 5 (45.45%) LN+ cases and 7 (24.14%) LN– cases, the right cranial was removed in 2 (18.18%) LN+ cases and 6 (20.69%) LN– cases, the right middle was removed in 1 (9.10%) LN+ cases and 5 (17.24%) LN– cases, the right caudal was removed in 2 (18.18%) LN+ and 10 (34.48%) LN– cases, and the accessory lung lobe was removed in 1 (9.10%) LN+ cases and 2 (6.90%) LN– cases. Eight LN+ (72.72%) cases had a single lung lobectomy performed and 3 (10.34%) had multiple lobes excised in order for all visible disease to be removed. For LN– cases, 24 (82.76%) had a single lung lobectomy and 5 (17.24) had greater than one lung lobe removed. Of the lymph nodes biopsied in LN+ cases, 4 (36.37%) were noted to be tracheobronchial, 1 (9.10%) was sternal, 2 (18.18%) mediastinal, 3 (27.27%) hilar and 1 (9.10%) was not specifically denoted. Of the LN– cases, 17 (58.26%) lymph node biopsies were noted to be tracheobronchial, 5 (17.24%) mediastinal, 7 (24.14%) hilar and 1 (3.45%) was not specifically identified. Three (10.34%) LN– dogs had more than one intrathoracic lymph node biopsied. For LN+ lymph node biopsies, only one lymph node per dog was noted to be biopsied in the records. The type of lymph node biopsy (incisional biopsy vs. excisional biopsy) was not specifically noted in the surgical reports and no tracheobronchial lymph nodes were biopsied from a median sternotomy approach.

### Histologic Variables

A summary of histologic diagnoses, mitotic index (MI), evidence of vascular or lymphatic invasion, grade, and completeness of surgical excision can be found in Table 2. The most common histologic diagnoses for the LN+ and LN– tumors were bronchoalveolar carcinoma [LN+ *n* = 4 (36.36%); LN– *n* = 21(72.41%)], and papillary pulmonary adenocarcinoma [LN+ *n* = 3 (27.27%); LN– *n* = 7 (24.14%)]. All tumors were identified histologically as primary lung tumors.

**Table 2:** Histologic findings as related to LN status.

	**LN+ (*n* = 11)**	**LN– (*n* = 29)**
**Histologic diagnosis**		
Tubulopapillary adenocarcinoma	1/11 (9.10%)	0/29
Bronchoalveolar carcinoma	4/11 (36.36%)	21/29 (72.41%)
Papillary pulmonary carcinoma	3/11 (27.27%)	7/29 (24.14%)
Adenosquamous carcinoma	2/11 (18.18%)	1/29 (3.45%)
Anaplastic carcinoma	1/11 (9.10%)	0/11
MI < 10	5/11 (45.45%)	12/29 (41.38%)
MI > 10	8/11 (72.72%)	13/29 (44.83%)
Vascular/lymphatic invasion	Yes 4/11 (36.36%)	Yes 5/29 (17.24%) No 17/29 (58.62%)
Intrabronchial seeding	1/11 (9.10%)	0
**Grade**		
I	0	3/29 (10.34%)
II	3/11 (27.27%)	14/29 (58.62%)
III	0	0
“High”	1/11 (9.10%)	0
Not reported	7/11 (63.64%)	12/29 (41.38%)
**Margins**		
Complete	4/11(36.36%)	27/32 (93.10%)
Incomplete	6/11 (54.55%)	0
Not reported	1/11 (9.10%)	2/29 (6.90%)

The mitotic index was available for 9 LN+ cases (81.82%) and in 27 LN– cases (84.38%). The (MI) was <10 for 1 (11.11%) LN+ cases and >10 for 8 (88.89%) LN+ cases. For LN– cases, the MI was available for 25 of 29 cases (86.20%). The MI was <10 for 12 (48.00%) and >10 for 13 (52.00%). Four of eleven (36.36%) LN+ dogs had evidence of vascular or lymphatic invasion, 1 (9.10%) had intrabronchial invasion, and there was no comment regarding vascular or lymphatic invasion for 6 (54.54%). For LN– dogs, 5 (17.24%) tumors had evidence of vascular or lymphatic invasion, 17 (58.62%) had none and there was no comment for 7 (24.14%). Regarding grade, there were 3 (27.27%) grade II pulmonary carcinomas in the LN+ dogs, 1 (691%) denoted as “high” and no grade provided for 7 (63.64%). For the LN– tumors, grade I was reported in 3 (10.34%), grade II was reported in 14 (48.28%) and there was no comment made for 12 (42.38%) tumors. Lung lobectomy surgical margins were reported as complete in 4 (36.36%) LN+ and 27 (93.10%) LN– dogs, incomplete in 6 (54.55%) LN+ and 0 LN–. No comment was made regarding surgical margins in 1 (9.10%) LN+ and 1 (3.45%) LN– cases and the margin was not inked in one LN– case (3.45%).

### Adjuvant Treatment

Four (36.36%) LN+ dogs received adjuvant MTD (Maximal Tolerated Dose) chemotherapy. Vinorelbine alone was used in 3 patients (8, 4 and 3 doses) and the fourth received 7 doses of vinorelbine, 6 doses of carboplatin and 2 doses of doxorubicin. Changes in chemotherapy protocols were based on evidence of progressive disease. Four (36.36%) LN+ dogs had surgery alone, one had surgery and prednisone (3.91%) and it could not be determined whether or not chemotherapy was administered in 2 dogs (18.18%).

For LN– dogs, 11/29 (27.59%) received adjuvant MTD chemotherapy, 12 (41.3.8%) did not receive chemotherapy and it was not known for 4 (13.79%) cases. Two of 29 (6.90%) LN– dogs received surgery and Prednisone. Vinorelbine was used as a single agent in 7/11 patients (63.64%); one dog received 5 doses, 3 dogs received 4 doses, and 2 dogs received 8 doses. One of 11 dogs received chlorambucil (9.10%), one dog received cyclophosphamide (9.10%), and one dog (9.10%) received piroxicam, doxycycline, and cyclophosphamide for six weeks and then vinorelbine (5 doses). Three (27.27%) LN+ dogs and 2 (6.90%) LN– dogs were lost to follow-up, and 3 (10.34%) LN– dogs were still alive at the time of writing.

### Survival Analyses

**Figure 1:**
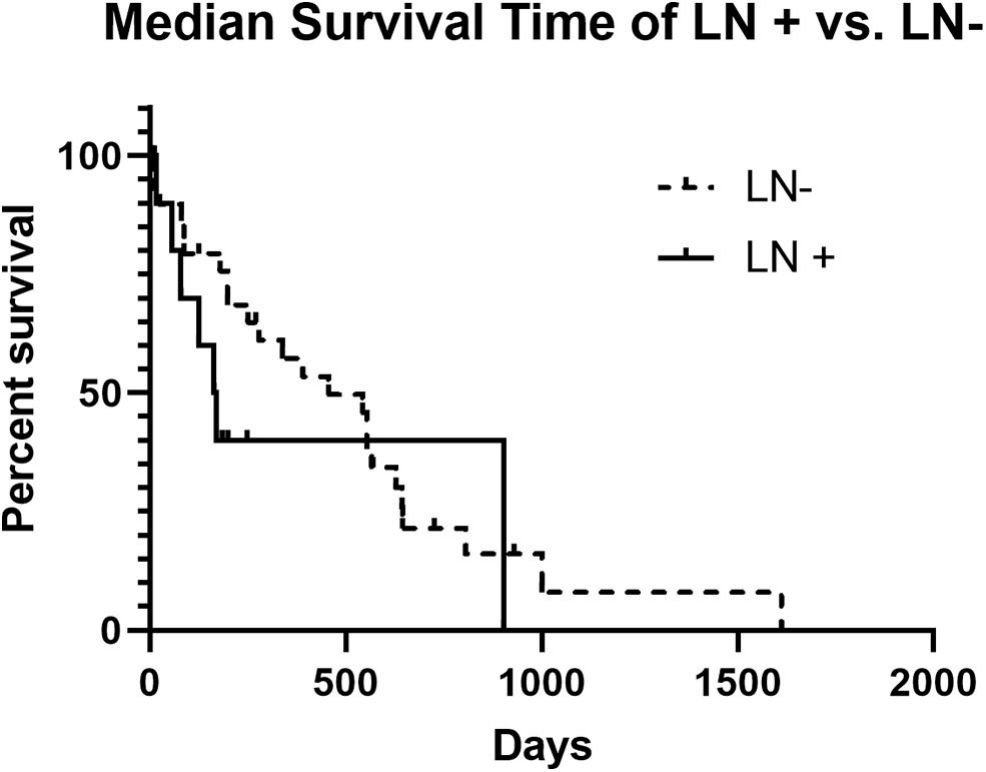
Kaplan-Meier Survival Curve Analysis of LN+ vs. LN– dogs. The median survival time of LN+ dogs was not significantly different than the median survival time for LN– dogs (167 days vs. 456 days and *p* = 0.2407).

**Figure 2:**
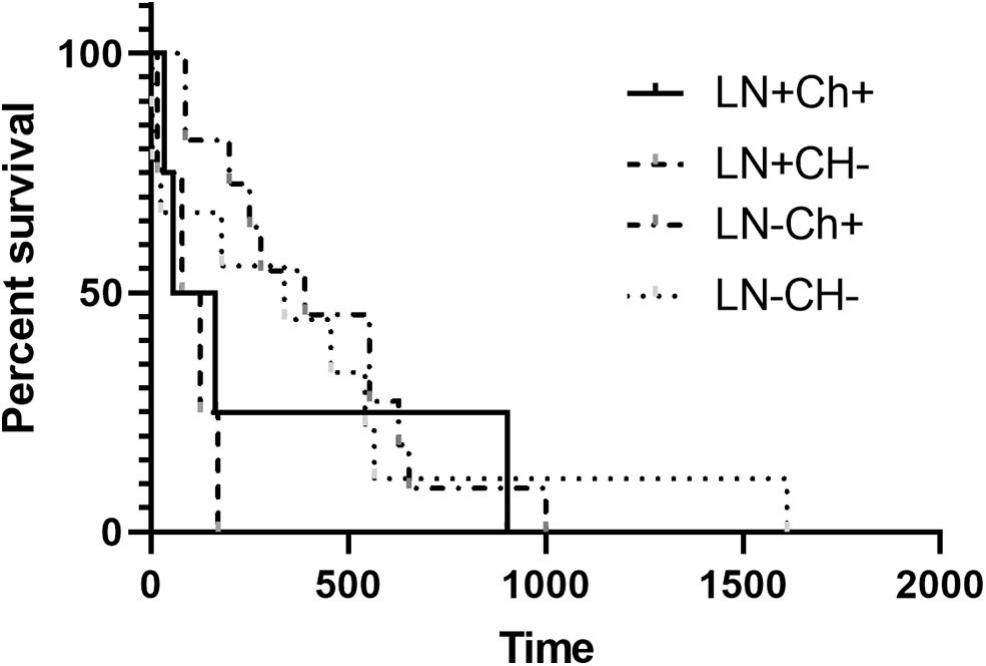
Kaplan-Meier Survival Curve Analysis of the median survival time with or without adjuvant chemotherapy in LN+ and LN– dogs. There was no difference in MST for dogs that were LN+ or LN– (*P* > 0.05).

The MST for LN+ dogs was 167 days, and for LN– dogs the MST was 456 days, which failed to be statistically different (*p* = 0.2407) (Figure 1). No significant difference in the MST in LN+ dogs was identified between dogs that received adjuvant chemotherapy (*n* = 4; 110 days) and those that did not receive adjuvant chemotherapy (*n* = 6; 125 days) (*p* = 0.4409) (Figure 2). There was no difference in survival time in LN– dogs receiving chemotherapy (*n* = 12; 335 days) as compared to those LN– dogs (*n* = 10) that did not receive adjuvant chemotherapy (258.5 days; *p* = 0.6475). There was no statistical difference in MST for patients that had a MI of <10 or >10 irrespective of lymph node status (*p* = 0.1096).

The MST between LN+ (*n* = 4) and LN– (*n* = 7) cases where tumor size was 101–999 cm^3^ was significantly different (110 and 198 days respectively; *p* = 0.0467; hazard ratio of 0.5556 with 95% CI of 0.1626–1.898). When evaluating LN– dogs, the MST of dogs with a tumor volume <100 cm^3^ (*n* = 5; MST 644d) was significantly longer than the MST of LN– dogs with a tumor volume between 100 and 999 cm^3^ (*n* = 7; MST 198d; *p* = 0.0232; hazard ratio of 3.164; 95% CI of 0.0928–1.079). No other statistical comparisons could be performed on the volume data due to no LN+ cases being in the <100 cm^3^ group, and one case in the LN+ and no LN– cases in the >1000 cm^3^ volume category.

The MST for LN+ dogs that received adjuvant MTD chemotherapy, not including Prednisone, (LN+CH+; *n* = 4) was 110 days and for those that did not receive adjuvant chemotherapy (LN+CH–; *n* = 4), the MST was 102 days wherein there was no significant difference (*p* = 0.6253). For LN– dogs, the MST for dogs that received chemotherapy (*n* = 11) was 390 days and for those that did not (*n* = 9), the MST was 338 days. Patients lost to follow-up and alive at the time of writing were censored prior to survival analysis. There was no difference in the MST for LN– dogs if they received adjuvant chemotherapy (*p* = 0.7192).

## Discussion

In this contemporary cohort of cases, the median survival of LN+ dogs was ~5.5 months, which is longer than previous reports where the MST ranged from 26–131 days although a direct comparison of MST cannot be performed and one could argue whether or not 131 days as compared to 167 days is clinically relevant (13, 21–23). However, this is an important finding to properly inform owners of outcome prior to treatment. The longer median survival time in this LN+ cohort of dogs may be explained by early identification of nodal metastasis on a microscopic level as compared to previous reports. The lack of LN enlargement on pre-operative CT in at least 3 of 11 LN+ dogs may support this theory. Similarly, it is being recognized that LN metastasis can occur with no alterations in LN size as is also seen for other canine neoplasms (28). It is strongly recommended that an intrathoracic LN biopsy be performed during lung lobectomy regardless of LN size as determined on CT imaging or intrathoracic assessments, to properly stage the patient and therefore recommend appropriate adjuvant treatment. In this case series, a minimum of 3 patients with metastatic disease would not have been identified had a LN biopsy not been performed based upon the LN size being noted as normal on CT imaging. Retrieval of intrathoracic lymph nodes, particularly the hilar and interlobar lymph nodes, is challenging even with understanding of canine anatomy yet critical for staging (29).

When evaluating the influence of tumor size, we identified that for LN– dogs, a tumor volume <100 cm^3^ had a longer survival than dogs where tumor sizes were noted to be between 101 and 999 cm^3^. As the majority of LN+ cases were within the 101–999 cm^3^, no other comparisons could be made and not surprisingly, dogs with LN+ and tumor size between 101 and 999 cm^3^ had a shorter survival time than LN– dogs with a similarly sized tumor.

We failed to reject our hypothesis that there will be no effect on survival with adjuvant chemotherapy for LN+ dogs. Given the low number of patients in this cohort, this data should be interpreted with caution as it may be reflective of type I or type II errors.

The limitations for this study include, low case numbers, possible errors in medical record reporting, histologic grading and re-evaluation of histologic surgical margins were not performed, and the study is retrospective in nature. Therefore, we did not evaluate the effect of tumor grade and completeness of excision on MST. Additionally, there was no standardized method for which intrathoracic lymph nodes were targeted for extirpation in relation to the anatomic lobar site of the excised primary tumor, nor were we able to decipher that the lymph node identified on CT as enlarged was the same lymph node that was biopsied intraoperatively. There was no standardized follow-up schedule utilized, and various chemotherapy agents were administered. A multi-center prospective case-controlled study should be performed to better define and understand the effect of treatment outcomes for dogs with primary pulmonary neoplasia and lymph node metastasis and the efficacy of adjuvant chemotherapy. Although no significant difference with chemotherapy was identified in this retrospective study, there were very few LN+ dogs to compare and this finding should be interpreted with caution.

In conclusion, we have identified a longer survival time in a contemporary series of dogs with primary pulmonary neoplasia with intrathoracic nodal metastasis. Surgical biopsy of intrathoracic lymph nodes during definitive tumor treatment by surgical excision is strongly recommended regardless of LN size on pre-operative imaging for appropriate staging.

## Data Availability Statement

The datasets generated for this study are available on request to the corresponding author.

## Ethics Statement

Ethical review and approval was not required for the animal study because this was a retrospective study of clinical patients receiving standard of care treatment at the veterinary teaching hospital. Written informed consent for participation was not obtained from the owners because this was a retrospective study of clinical patients receiving standard of care treatment at the veterinary teaching hospital.

## Author Contributions

RR and DW contributed equally to the production of this manuscript. Both authors contributed to the article and approved the submitted version.

## Conflict of Interest

The authors declare that the research was conducted in the absence of any commercial or financial relationships that could be construed as a potential conflict of interest.

## Footnotes

^1^GraphPad Prism 7 (GraphPad Software, San Diego, CA).

^2^IBM SPSS Statistics 2019 (Armonk, NY).
